# Supplementary material for: Curated and harmonised transcriptomics datasets of interstitial lung diseases
Source: Data Brief. 2025 Oct 14;63:112139. doi: 10.1016/j.dib.2025.112139 (PMC12581653; doi:10.1016/j.dib.2025.112139)
Supplement: Supplementary file 1 [file mmc1.zip › Supplementary_material/RNA-seq/fastQC reports/GSE184316/TRIMMED_SRR15926776_fastqc.html]

TRIMMED\_SRR15926776.fastq.gz FastQC Report 

FastQC Report

Sat 3 Dec 2022  
TRIMMED\_SRR15926776.fastq.gz

## Summary

- Basic Statistics
- Per base sequence quality
- Per sequence quality scores
- Per base sequence content
- Per sequence GC content
- Per base N content
- Sequence Length Distribution
- Sequence Duplication Levels
- Overrepresented sequences
- Adapter Content

## Basic Statistics

| Measure | Value |
| --- | --- |
| Filename | TRIMMED\_SRR15926776.fastq.gz |
| File type | Conventional base calls |
| Encoding | Sanger / Illumina 1.9 |
| Total Sequences | 5485378 |
| Sequences flagged as poor quality | 0 |
| Sequence length | 35-371 |
| %GC | 51 |

## Per base sequence quality

## Per sequence quality scores

## Per base sequence content

## Per sequence GC content

## Per base N content

## Sequence Length Distribution

## Sequence Duplication Levels

## Overrepresented sequences

| Sequence | Count | Percentage | Possible Source |
| --- | --- | --- | --- |
| CACCTCCCCGAAGCTCAGGGAGAGCCCTGTTAGGGCCGCCTCTGGCCCTA | 97337 | 1.7744811752262106 | No Hit |
| CAGTGGGGCCACGAGCTGAGTGCGTCCTGTCACTCCACTCCCATGTCCCT | 86976 | 1.5855972004117127 | No Hit |
| AGCTGCTAAGTGCTGTGTTGTCGTTCCCCCTGCTTAAAATAAAGTTGTTT | 43744 | 0.79746555296645 | No Hit |
| CCCTGGCTGCTACAGGAGAATAGCAGACAGGTATAGTTAAGAAACAACTT | 42497 | 0.7747323885427768 | No Hit |
| CAGCAGTTTACACCTGCTAGGGTGTTCAAAGGTCAGTGCTATAGAAATTC | 41277 | 0.7524914417930725 | No Hit |
| AACAAGCACAAAGCCAAGAAAACCAACGATGCCAGATACTGAATTTCTAT | 32996 | 0.6015264581583986 | No Hit |
| AGACCACCAGCAGCCTGCCCAGGGCCTCACCACCAACTTC | 29005 | 0.5287693938321115 | No Hit |
| GCCGACAAGACCAACGTCAAGGCCGCCTGGGGTAAGGTCGGCGCGCACGC | 28926 | 0.527329201378647 | No Hit |
| AGGTCTTGGTGGTGGGGAAGGACAGGAACATCCTCTCCAGGGCCTCCGCA | 26295 | 0.47936532359301404 | No Hit |
| GAAGTTGGTGGTGAGGCCCTGGGCAGGCTGCTGGTGGTCT | 23723 | 0.43247703257642406 | No Hit |
| AGAGGGCTCCCAGAAGTGTGTGGCTGAGCTGGGTCCCCAGGCCGTGGGGG | 22065 | 0.40225122133789143 | No Hit |
| CCAGTCTCGGCTCAGCCAAACACTGTCAGGGCCCCCAGCAGGGCCTTCAG | 21219 | 0.3868284008868669 | No Hit |
| TGATATGGTTCCTGGCAAGCCCATGTGTGTTGAGAGCTTCTCAGACTATC | 18753 | 0.3418725199977103 | No Hit |
| GAGTGCGCTGCACTGACCTTCTTCCAAGCCTCAGTTCCTGTTCTAGGAAC | 18543 | 0.33804416031128576 | No Hit |
| CAATCCACTTGATCCCAACTCATCTCTCATTTATTTCGGCTTCTTTTATT | 18247 | 0.33264799618184926 | No Hit |
| AGGCTACACACTGAGGACTCTGTTCCTCCCCTTTCCGCCTAGGGGAAAGT | 18087 | 0.3297311507064782 | No Hit |
| GCCAACACACAAGCAGTTCACAGTAGAACACTGCAGACTGCAGGGCATTT | 17588 | 0.3206342388801647 | No Hit |
| TGATGACACCCACCGCAACTGTCTGTCTCATATCACGAACAGCAAAGCGA | 17563 | 0.3201784817746379 | No Hit |
| TTCAGCCCTGATCAAGACATGAGGGAGGCAGGGGCTCAGCTGAAGAAGCT | 17023 | 0.31033412829526064 | No Hit |
| GGAAGACAGTGTGGCGATTCCTCAGGGATCTAGAACTAGAAATACCATTG | 16855 | 0.30727144054612093 | No Hit |
| GCAGCTGCTTCCGCATAGCTGCTGTGGTCAAAAAGGAGCCCAGAGTGACA | 16607 | 0.3027503300592958 | No Hit |
| TGGGTCTGCGGTGACCCTATGCATTCCTTCAGTGCTTGCTAGAACAGTTT | 16326 | 0.2976276201931754 | No Hit |
| TCTTGGGTTATAATCATTGGCAATGTTGTAATCAACAACCCATGTTATTT | 16004 | 0.2917574686739911 | No Hit |
| ATTGATTCTTCCTACCCATGAGCATGGAATGTTCTTCCATTTGTTTGTAT | 14939 | 0.2723422159785524 | No Hit |
| ACAACAAACAGAACGGCGACCGTCAAGGAAAACTGTCACTCTGGGCTCCT | 14328 | 0.26120351231947914 | No Hit |
| GAAGGACCTCTTCAAGGAGAACTACAAACCACTGCTCAAGGAAATAAAAG | 14211 | 0.2590705690656141 | No Hit |
| GGATGGAGCAGGGCAAGGCCTCAAACCGTTTCAAAACTGTTCTAGCAAGC | 13483 | 0.24579892215267574 | No Hit |
| TTACAAAAGCAGAATAAAAGCGAAAAGAAATGAAAATGTTACACTACATT | 13432 | 0.24486917765740118 | No Hit |
| GGAAAGGGGGCACTGACTGGTGATGCTGATATTGGAGTGCATCTGAGGCT | 12778 | 0.23294657177682196 | No Hit |
| GGGAAGCGGGGAATGTCTACGTGCGTATGCACGTGGCACTCTCTGCCCGA | 12585 | 0.2294281269221556 | No Hit |
| GACGAGTCAGAAAGGTCACGTCCTGCTCTTGGTCCTTGTCAGTGCCATGT | 11670 | 0.21274741685987728 | No Hit |
| CACTGTTTCTTATAGCGGTGGTTTAAACAGAGGTGCAAACAGCAAGCGGA | 11205 | 0.20427033469708014 | No Hit |
| ATAGCAGCATGATTTATAGTCCTTTGGGTATATACCCAGTAATGGGATGG | 10534 | 0.1920378139847427 | No Hit |
| CAATAAATAGGACACTTCTGCCAAAGGAACTCGTGCACAGCACCACAGAA | 10490 | 0.19123568147901565 | No Hit |
| AGGCCCCAAAAGAAAACAGCGTCTTACTGGGGAGGGATATGTGCTTTCTC | 10297 | 0.1877172366243493 | No Hit |
| TTTTTCCATGAGCTTAATGATGCTTTCTCTGGGCTTTTGGGGGAGGGTGT | 9574 | 0.1745367411325163 | No Hit |
| GAAGGACCTCTTCAAGGAGAACTACAAACCACTGCTCAATGAAATAAAAG | 9550 | 0.17409921431121064 | No Hit |
| CTTCATGATGTAGCAGCAGGTGCCAGGGGCTGGCTTGTAGGCGATCAGCA | 8950 | 0.16316104377856913 | No Hit |
| TACCACTGCCACCTTCTCCATCGGCTCCACTGGCCTCGTGGTGTATGACT | 8584 | 0.1564887597536578 | No Hit |
| GGCAATGAGCGGTTCCGCTGCCCTGAGGCACTCTTCCAGCCTTCCTTCCT | 8505 | 0.15504856730019337 | No Hit |
| GTCCACGTCACACTTCATGATGGAGTTGAAGGTAGTTTCGTGGATGCCAC | 8041 | 0.14658971542161725 | No Hit |
| GGCGCAGGGGTACGGACCTCAGCTGAGTCATGGGAGCTGAATGTATGTGT | 7989 | 0.14564174064212165 | No Hit |
| CCAGGGACTTTTTGTTGCCTGTAAAGTGCTCTGGCATTGCCTGAGGATAG | 7942 | 0.1447849172837314 | No Hit |
| TTTTCCATGAGCTTAATGATGCTTTCTCTGGGCTTTTGGGGGAGGGTGTC | 7550 | 0.1376386458690723 | No Hit |
| CAATGGGGTACTTCAGGGTCAGGATGCCACGCTTGCTCTGGGCCTCGTCG | 7087 | 0.12919802427471724 | No Hit |
| CAAGGAAAGAGAGGCCACAGTAAGGCCCATCCGAGGTCCTGAGGAGATCT | 7019 | 0.12795836494768456 | No Hit |
| AGTTGCACTTGGCTTCACCCGTGTGACTTTCGTAACGGGGAGAGAGAGAA | 6995 | 0.12752083812637888 | No Hit |
| TGAGGGGCACGGCCACAGCCCCCCAAAGGCGACAAGATCCGCTTGCTGTT | 6991 | 0.12744791698949462 | No Hit |
| GTCTCAACATCTGTGTTGGGGAGAGTGGAGACAGACTGACGCGAGCAGCC | 6325 | 0.11530654769826255 | No Hit |
| CAGCCGTGGCAGTCTCCCTTCCTAGCCATGGAAGAGCATATCCTTGTTTA | 6190 | 0.1128454593284182 | No Hit |
| AGACCACCAGCAGCCTGCCCAGGGCCTCACCACCAACTTCATCCACGTTC | 6096 | 0.11113181261163771 | No Hit |
| GTGTCCAGAGACAACACTAGACCAGTACTATGATCCCAAGTTACTCTTTG | 5962 | 0.10868895452601443 | No Hit |
| GCAATGTAGGGGCCGGTGGACCTGCTCCAGCAGCTGGTGCTGCACCAGCA | 5785 | 0.10546219421888518 | No Hit |
| ACCTCTCTCTGGGCTTCTATTTCGACCGCGATGATGTGGCTCTGGAAGGC | 5774 | 0.10526166109245341 | No Hit |
| CTGACAGTGTATCTAGCTTTGGAAAACACAGGGGTCTGCCCTGTGAGCTG | 5644 | 0.10289172414371445 | No Hit |
| ATAGCAGCATGATTTATAATCCTTTGGGTATATACCCAGTAATGGGATGG | 5641 | 0.10283703329105123 | No Hit |
| GGTAGCAGTGTCTGTCTGTGTTTTGCATTCAGATCTTGCTATCCACACAA | 5529 | 0.10079524145829148 | No Hit |

## Adapter Content

Produced by FastQC (version 0.11.7)
